# A highly selective, orally active inhibitor of Janus kinase 2, CEP-33779, ablates disease in two mouse models of rheumatoid arthritis

**DOI:** 10.1186/ar3329

**Published:** 2011-04-21

**Authors:** Kristine L Stump, Lily D Lu, Pawel Dobrzanski, Cynthia Serdikoff, Diane E Gingrich, Ben J Dugan, Thelma S Angeles, Mark S Albom, Mark A Ator, Bruce D Dorsey, Bruce A Ruggeri, Matthew M Seavey

**Affiliations:** 1 Cephalon, Inc., Research Drug Discovery, 145 Brandywine Parkway, Building 200, West Chester, PA 19380-4249, USA

## Abstract

**Introduction:**

Janus kinase 2 (JAK2) is involved in the downstream activation of signal transducer and activator of transcription 3 (STAT3) and STAT5 and is responsible for transducing signals for several proinflammatory cytokines involved in the pathogenesis of rheumatoid arthritis (RA), including interleukin (IL)-6, interferon γ (IFNγ) and IL-12. In this paper, we describe the efficacy profile of CEP-33779, a highly selective, orally active, small-molecule inhibitor of JAK2 evaluated in two mouse models of RA.

**Methods:**

Collagen antibody-induced arthritis (CAIA) and collagen type II (CII)-induced arthritis (CIA) were established before the oral administration of a small-molecule JAK2 inhibitor, CEP-33779, twice daily at 10 mg/kg, 30 mg/kg, 55 mg/kg or 100 mg/kg over a period of 4 to 8 weeks.

**Results:**

Pharmacodynamic inhibition of JAK2 reduced mean paw edema and clinical scores in both CIA and CAIA models of arthritis. Reduction in paw cytokines (IL-12, IFNγ and tumor necrosis factor α) and serum cytokines (IL-12 and IL-2) correlated with reduced spleen CII-specific T helper 1 cell frequencies as measured by *ex vivo *IFNγ enzyme-linked immunosorbent spot assay. Both models demonstrated histological evidence of disease amelioration upon treatment (for example, reduced matrix erosion, subchondral osteolysis, pannus formation and synovial inflammation) and reduced paw phosphorylated STAT3 levels. No changes in body weight or serum anti-CII autoantibody titers were observed in either RA model.

**Conclusions:**

This study demonstrates the utility of using a potent and highly selective, orally bioavailable JAK2 inhibitor for the treatment of RA. Using a selective inhibitor of JAK2 rather than pan-JAK inhibitors avoids the potential complication of immunosuppression while targeting critical signaling pathways involved in autoimmune disease progression.

## Introduction

Inflammation acting as a driver of pathology has previously been thought to be primarily restricted to infectious diseases and autoimmune disorders; however, it has become more apparent that inflammation plays a larger role in multiple different disease indications, such as obesity, coronary artery disease and cancer [1,2]. Targeting mediators of inflammation has received enormous attention in the past decade with the advent of biologics, most notably antibodies that capture or neutralize disease-driving cytokines [3]. However, biologics have several disadvantages compared with orally active small molecules, such as route of administration, cost of production and the possibility of anaphylactic reactions [4]. Unlike the direct targeting of cell surface receptors or cytokines, another approach is to target cytokine pathways by inhibiting key kinases involved in transducing cytokine receptor signals [5]. In the present article, we describe the preclinical evaluation of a potent, orally active, small-molecule inhibitor of Janus kinase 2 (JAK2), CEP-33779, for the treatment of rheumatoid arthritis (RA).

RA is a chronic systemic inflammatory disorder that primarily affects the synovial joints. RA can also produce diffuse inflammation in the lungs and pleura and also forms subcutaneous nodular lesions. About 1% to 2% of the world's population is affected by RA, and there is a gender bias associated with disease onset. Women are three times more likely than men to develop RA between the ages of 35 and 50 years. Treatments for RA have included mainly disease-modifying antirheumatic drugs and have been more prolific than those for the more difficult to treat rheumatic diseases, such as lupus. Approved treatments for RA include nonsteroidal anti-inflammatory drugs, antimetabolites such as methotrexate and leflunomide, various corticosteroids and glucocorticoids, sulfasalazine, and various biologics, including abatacept, adalimumab, etanercept, infliximab, golimumab, and rituximab [3]. Other molecules implicated in the pathogenesis of RA include B lymphocyte stimulator/B cell activating factor, a proliferation-inducing ligand, p38/mitogen-activated protein kinase, and the B-cell receptor protein spleen tyrosine kinase Syk [6,7]. The recent US Food and Drug Administration approval of tocilizumab (anti-interleukin (IL)-6R) further demonstrates the power of targeting cytokines and associated receptors to treat chronic inflammatory diseases [8].

Using small molecules to target cytokine pathways is an attractive method to treat disease simply because of the oral bioavailability of small molecules and the reduced cost of production, thus reducing the cost carried over to the patient and healthcare system as a whole. The safety profile of antibody biologics, especially anti-tumor necrosis factor α (anti-TNFα) antibodies, has come under greater scrutiny over the past several years as more and more patients and physicians have reported problems associated with long-term use [9-11]. It is well-documented that JAK kinases play a pivotal role in cytokine receptor signaling to phosphorylate and activate signal transducer and activator of transcription (STAT) proteins. Several of these JAK-controlled cytokine receptor pathways are intimately involved in the initiation and progression of RA disease pathogenesis [12]. Cytokines involved in these diseases (for example, interleukin (IL)-2, IL-6, IL-12, interferon γ (IFNγ) and granulocyte macrophage colony-stimulating factor (GM-CSF)) are also essential to a properly functioning immune response to infectious agents. JAK1, JAK2 and tyrosine kinase 2 (TYK2) kinases are ubiquitously expressed, whereas JAK3 is limited to the lymphoid lineage as JAK3 associates with the common γ-chain.

The therapeutic benefit of JAK kinase inhibition has already been established in RA with the use of CP-690,550, a pan-JAK inhibitor originally intended for organ transplantation immunosuppression, as it is a potent inhibitor of JAK3 but has also been shown to have activity against JAK1 and JAK2 [13]. More recently, the selective JAK1/JAK2 inhibitor INCB028050 has been shown to have efficacy in various rodent models of RA, further demonstrating the central role that JAK kinase plays in this disease [14]. The mechanistic rationale of targeting cytokine pathways central to the pathogenesis of RA, but without the immunosuppressive side effects of several of the TNFα blockade biologics, has been demonstrated in the successful use of tocilizumab to treat RA [15]. The present report shows that a novel, potent, orally active, selective JAK2 inhibitor, CEP-33779, can ameliorate disease in two mouse models of RA. JAK2 inhibition decreased several inflammatory cytokines, including IL-6, IL-12, IL-1β and IFNγ, both systemically and locally at the site of disease and treated symptoms of chronic, pathological joint destruction in a well-tolerated manner.

## Materials and methods

### Animals, antibodies, cell culture reagents, compounds, cell lines

Mice used for collagen-induced arthritis (CIA) and collagen-antibody induced arthritis (CAIA) studies were Harlan female DBA/1 mice (Harlan, Somerville, NJ, USA) Female Balb/c mice from Jackson Laboratories (Bar Harbor, ME, USA) were used for all air pouch model (APM) studies. Mice were age-matched 6 to 8 weeks from the start of the experiments. All animals were maintained on a 24-hour light-dark cycle, with food and water available *ad libitum*. All experimental animal procedures were approved by, and in accordance with the regulations of, the Institutional Animal Care and Use Committee of Cephalon, Inc.

### Compound

CEP-33779 was prepared in a manner analogous to the five-step method described elsewhere [16]. Compound CEP-33779 was administered orally in suspension form, dissolved in dimethyl sulfoxide (DMSO) (1% final concentration) (Sigma, St. Louis, MO, USA), and further reconstituted in PEG400 (Emerald BioSystems, Bainbridge Island, WA, USA) to the desired concentration for oral administration. The compound was administered orally through gavage tubes for all animals treated with CEP-33779.

All spleen samples were analyzed using an Accuri C6 Flow Cytometer (Accuri Cytometers, Ann Arbor, MI, USA). Antibodies used for Western blot analysis were anti-phosphorylated STAT5 (Cell Signaling Technology, Danvers, MA, USA) and anti-total STAT5 (Cell Signaling Technology). Complete media (R10) was used for all experiments involving the *ex vivo *culture of splenocytes for all enzyme-linked immunosorbent spot assay (ELISPOT) experiments. Complete media consisted of RPMI 1640 (Cellgro, Manassas, VA, USA) plus 1% Pen-Strep (Cellgro), 1% L-Gln (Cellgro), 1% nonessential amino acids (Cellgro), β-mercaptoethanol (Sigma), and 10% fetal bovine serum (Cellgro). The small-molecule JAK2 inhibitor CEP-33779 was synthesized according to procedures detailed elsewhere [16]. The cell line HEL92 (American Type Culture Collection, Manassas, VA, USA) was used for early pharmacodynamic (PD) testing of phosphorylated STAT5 (pSTAT5) inhibition using CEP-33779.

### Western blot analysis

Samples were heated at 70°C for 10 minutes and loaded onto a NuPAGE 10% Bis-Tris gel (1.5 mm, 15 wells; Invitrogen, Carlsbad, CA, USA). The molecular weight marker used was a Bio-Rad kaleidoscope prestained standard (Bio-Rad, Hercules, CA, USA). Run conditions were 150 V for 1 hour at room temperature (RT). Using a semidry transfer apparatus (Bio-Rad), proteins were transferred onto a nitrocellulose membrane. Primary antibody diluted at 1:1,000 in 5% milk for pSTAT3 pY705 (Cell Signaling Technology) and total STAT3 (Cell Signaling Technology) diluted to 1:1,000 in 5% bovine serum albumin (BSA) in Tris-buffered saline (TBS) were incubated overnight at 4°C with 5% milk in TBS plus 0.05% Tween 20 (TBS-T). Anti-rabbit immunoglobulin G (IgG), horseradish peroxidase (HRP)-linked secondary antibody was used at 1:2,000 dilution (Cell Signaling Technology). Detection was performed using SuperSignal West Pico Chemiluminescent Substrate (Pierce Biotechnology, Rockford, IL, USA). Densitometry was performed using Gel-Pro Analyzer 3.1 software (Media Cybernetics, Inc., Bethesda, MD, USA).

### Enzyme assays

The kinase activity of baculovirus-expressed human JAK kinases (JAK1, JAK2, JAK3 or TYK2) was measured using the time-resolved fluorescence detection system as described elsewhere [17]. Each 96-well Costar high binding plate (Corning, Corning, NY, USA) was coated with 100 μl/well of 10 μg/ml neutravidin (Pierce Biotechnology) in TBS at 37°C for 2 hours, followed by 100 μl/well of 1 μg/ml 15-mer peptide substrate (biotinyl-amino-hexanoyl-EQEDEPEGDYFEWLE-amide; Infinity Biotech Research and Resource, Aston, PA, USA) at 37°C for 1 hour. The kinase assay mixture (total volume 100 μl/well), consisting of 20 mM 4-(2-hydroxyethyl)-1-piperazineethanesulfonic acid (pH 7.2), adenosine triphosphate (ATP) (*K*_m _level for each kinase), 1 mM MnCl_2_, 0.1% BSA, and test compound (diluted in DMSO; 2.5% DMSO final concentration in assay), was added to the assay plate. The concentrations of ATP used were as follows: 0.2 μM ATP for JAK1, JAK2, and TYK2 and 0.1 μM ATP for JAK3. Enzyme was added, and the reaction was allowed to proceed for 20 minutes at RT. Detection of the phosphorylated product was performed by adding 100 μl/well of Eu-N1-labeled PY100 antibody (PerkinElmer Life Sciences, Boston, MA, USA) diluted 1:5,000 or 1:10,000 in 0.25% BSA in TBS-T. Samples were incubated at RT for 1 hour, followed by the addition of 100 μl of enhancement solution (PerkinElmer Life Sciences). Plates were agitated for 10 minutes, and the fluorescence of the resulting solution was measured using the PerkinElmer EnVision 2102 or 2104 multilabel plate reader. Half maximal inhibitory concentration (IC_50_) values were determined using the four-parameter logistic model in XLFit 4 (IDBS, Ltd., Guildford, UK).

### CAIA model

Acclimated mice were randomized, prebled and measured for baseline inflammation before any studies were initiated. On day 0, DBA/1 female mice were injected intravenously (i.v.) with 100 μl of a saline solution containing 1.5 mg of a 10 mg/ml cocktail of arthritogenic monoclonal antibodies directed against different epitopes of CII (Chondrex, Redmond, WA, USA) suspended in saline solution. On day 3, mice were injected intraperitoneally (i.p.) with 100 μl of 50 μg of *Escherichia coli *0111:B4 lipopolysaccharide (LPS) (0.5 mg/ml stock) (Chondrex) and fed gel food during recovery. Two days after LPS treatment monitoring of arthritic paws began. Peak arthritis usually occurred 2 to 3 days following LPS treatment. To ensure that enough mice were available for each study, a surplus of animals were induced, and only those mice that met the criteria were entered into the study. Mice that exhibited a clinical score greater than 1 in each limb were considered arthritic and were entered into the study. If mice did not meet the study criteria, they we excluded and killed. Mice were then randomized and ear-tagged for identification. Study group sizes were determined after study entry, but consisted of at least 10 mice per group. Arthritis clinical score tables can be found elsewhere [18]. Briefly, a score of 0 denotes no evidence of erythema and swelling, 1 denotes erythema and mild swelling confined to the tarsus, 2 denotes erythema and mild swelling extending from the ankle to the midfoot, 3 denotes erythema and moderate swelling extending from the ankle to the metatarsal joints, and 4 denotes erythema and severe swelling encompassing the ankle, foot and digits. The black arrows in the respective figures indicate treatment start. CEP-33779 compound was administered orally (p.o.) by gavage instrument suspended in vehicle twice daily (b.i.d.). Vehicle consisted of PEG400 plus 1% DMSO administered p.o., b.i.d., and dexamethasone (Dex) (Hanna's Pharmaceutical Supply, Wilmington, DE, USA) at 1.5 mg/kg was administered i.p. three times weekly in saline solution and continued from the peak of inflammation until the end of the experiment. Mice were cheek-bled for serum sample collection for cytokines and antibodies throughout the experiment. All samples were kept at -80°C until ready to assay. At about 4 weeks, mice were harvested for spleen, serum and arthritic paws for STAT and cytokine quantitation and histological analysis. For earlier validation, studies were ended around 3 to 4 weeks after induction, and the paws were removed at various time points to measure cytokine levels over time.

### CIA model

Acclimated mice were randomized, prebled and measured for baseline inflammation before any studies were initiated. On day 0, DBA/1 female mice were given a primary immunization with equal volumes of bovine CII, 2 mg/ml stock in 0.05 M acetic acid (Chrondrex) suspended in 5 mg/ml Complete Freund's Adjuvant (CFA) (Chondrex) emulsion, yielding an injection of 100 μg of CII at 100 μl into the base of the tail intradermally (i.d.). At day 21 post-primary immunization, mice were rechallenged with CII emulsified in Incomplete Freund's Adjuvant (IFA) (Thermo Scientific, Rockford, IL, USA) subcutaneously (s.c.) on the flank of the mouse. On day 28, each mouse received an i.p. injection of *E. coli *0111:B4 LPS (0.5 mg/ml stock) (Chondrex) at 10 μg in 100 μl of saline solution (25 to 50 μg of LPS reduced to 10 μg per mouse to reduce mortality associated with endotoxic shock). Around day 35 the mice begin to show signs of paw inflammation and disease. Before the start of treatment, mice were randomized, grouped, scored, ear-tagged and prebled to determine baseline disease. To ensure that enough mice were included in each study, a surplus of animals were induced, and only those mice that met the study criteria were entered into the study. Mice that exhibited a clinical score greater than 1 in each limb were considered arthritic and were entered into the study. If mice did not meet the study criteria, they were excluded and killed. Mice were then randomized and ear-tagged for identification. Study group sizes were determined after study entry, but consisted of at least 10 mice per group. Arthritis clinical scores were the same as those used to score the mice in the CAIA model (see CAIA model). The black arrows in the respective figures indicate treatment start. CEP-33779 compound was administered orally by gavage suspended in vehicle b.i.d. Vehicle consisted of PEG400 plus 1% DMSO p.o., b.i.d., and 1.5 mg/kg Dex (Hanna's Pharmaceutical Supply) was administered i.p. three times weekly in saline solution and continued from the peak of inflammation until the end of the experiment. Mice were cheek-bled for serum sample collection for cytokines and antibodies throughout the experiment. All samples were kept at -80°C until ready to assay. At about day 60, mice were harvested for spleen, plasma and arthritic paws for STAT and cytokine quantitation and histological analysis.

### APM for carrageenan-induced inflammation

Details on the establishment and design of this rapid inflammation screening model can be found elsewhere [19,20]. Briefly, Balb/c mice were injected s.c. with 4 ml of sterile air on day -5, baseline measurements were recorded, and mice were randomized into groups. Mice entered the study upon air pouch maintenance by day -3, when 2 ml of sterile air was injected for air pouch maintenance. On day 0, mice were given a 1% carrageenan (Sigma) injection suspended in saline solution directly into the air pouch. This induced a rapid inflammatory influx of primarily neutrophils and macrophages (data not shown). Compound was administered in saline solution at milligrams per kilogram concentrations in 100 μl total volume directly into the air pouch simultaneously with carrageenan injection (1% carrageenan in saline solution). The response was followed by a spike in IL-6, TNFα, and IL-1β pouch cytokines (data not shown) and was completed by day 8 (data not shown). Twenty-four hours after carrageenan and compound administration, 100 μl of air pouch fluid was removed along with cellular exudates. Cellular exudates were centrifuged, the supernatants were processed for Luminex cytokine analysis (Invitrogen), and the cell pellets were counted for total live and dead cells. Total cell counts were performed using BD TruCount beads (BD Biosciences, San Jose, CA, USA), and cellular analysis was performed using 7-aminoactinomycin D (Sigma) as a viability dye to exclude dead cells. Hemoglobin content was determined using the Drabkin's assay (Sigma).

### Histology

#### CAIA and CIA arthritis models

At the end of each experiment, front and hind paws (including carpus and tarsus) were removed from the body of each animal for histological analysis. Skin from the ends of the digits was removed, and the metatarsal region skin was perforated using surgical scissors to allow full decalcification. Samples were treated using a decalcification procedure with formic acid as described elsewhere [21,22]. After 7 to 10 days of decalcification, samples were washed for 2 hours in distilled water and stored in 70% ethanol at 4°C until they were ready to be processed. Samples were paraffin-embedded, sectioned, and stained with hematoxylin and eosin as well as Safranin O for matrix degradation (images not shown, used by pathologist for scoring). All histological work was performed at the Wistar Institute (Philadelphia, PA, USA). Images were collected using an Olympus BX50 microscope equipped with an Olympus DP70 camera and Olympus LabSens software (Olympus, Center Valley, PA, USA). A total of five mice from each group were analyzed via histopathology, and representative × 10 original magnification images are shown for each group tested.

### Measurement of serum anti-collagen type I and anti-CII autoantibodies

Serum was collected every 1 to 2 weeks and stored at -80°C until use. Thawed samples were analyzed by using an in-house-generated CII and collagen type I (CI) enzyme-linked immunosorbent assay (ELISA) method. Briefly, each well of a 96-well plate was coated with 50 μl of 5 μg/ml CI (Chondrex) or CII (Chondrex) in borate-buffered saline (BBS) (0.025 M Na_2_B_4_O_7_-10H_2_O, 0.01 M H_3_BO_3_, and 0.075 M NaCl; pH 8.4) buffer overnight at 4°C. Plates were washed with BBS plus 0.1% Tween 20 three times before proceeding. Standard curves were generated using purified mouse anti-CI or anti-CII antibody, and serum was added at 50 μl per well and then incubated at RT for 1 hour followed by being washed with BBS plus Tween 20. After being washed, 50 μl of 1 μg/ml rabbit anti-mouse HRP-fragment antigen-binding antibody was added as a detection antibody (Rockland Biologicals, Gilbertsville, PA, USA) and incubated at RT for 45 minutes. Plates were washed four times with BBS plus Tween 20, then 100 μl of tetramethylbenzidine substrate was added and the reaction was stopped using 100 μl of 1 M H_2_SO_4_. The plates were read at 450 nm with a reference wavelength of 570 nm.

### ELISPOT assays

Descriptions of T-cell and B-cell ELISPOT methods can be found elsewhere [23,24]. The technique used for each is described briefly here.

#### T-cell ELISPOT assays

For CIA experiments, Millipore nitrocellulose IP filter plates (Millipore, Billerica, MA, USA) were coated with either anti-mouse IFNγ (AN18; MabTech, Mariemont, OH, USA) or anti-mouse IL-4 (11B11; eBioscience, San Diego, CA, USA) at 7 μg/ml or 2 μg/ml, respectively, in sterile phosphate-buffered saline (PBS) at 60 μl/well and incubated for 3 hours at RT or overnight at 4°C. Coated wells were washed with PBS plus 0.1% Tween 20 and patted semidry before blocking using complete media for at least 1 hour before use. Purified mouse CII (Chrondrex), CI (Chrondrex), or third-party control chicken ovalbumin (OVA) (Sigma) was used to challenge 1 million processed splenocytes per well at 10 μg/ml total antigen concentration. Media alone was used as a background control, OVA was used as a third-party control, and phorbol 12-myristate 13-acetate plus ionomycin was used as a positive control for IFNγ and IL-4 release by T helper 1 (Th1) and Th2 cells, respectively.

#### B-cell ELISPOT assays

B-cell ELISPOT components were obtained from MabTech, and nitrocellulose IP filter plates were obtained from Millipore. Briefly, for CIA experiments, B-cell ELISPOT wells were activated using ethanol, then washed and coated with 10 μg/ml purified bovine CII or purified bovine CI (Chrondrex) in sterile PBS. OVA was used as a third-party control, and media alone was used for background. Processed splenocytes were added to each well and were not stimulated with LPS to avoid true *ex vivo *frequencies of antibody-secreting cell types (ASCs). Anti-mouse total IgG was used as a positive control for total IgG-producing ASCs and was used to normalize results. Cell frequencies for the detection of each antigen were previously identified during optimization and validation phases for each model tested (data not shown). A total of 500,000 cells were added to each well for the CI- and CII-coated wells for CIA experiments. B-cell ELISPOT spots were incubated overnight at 37°C and 5% CO_2_. To develop each assay, a secondary antibody (MabTech) was added to each well, incubated, and washed; alkaline phosphatase streptavidin was used as a conjugate (Jackson Immunoresearch, West Grove, PA, USA); and 5-bromo-4-chloro-3'-indolylphospate ρ-toluidine salt (BCIP) and nitro-blue tetrazolium chloride (NBT) (Rockland Biologicals) was used as a substrate and developed until spots were visible. All ELISPOT analyses were performed using a CTL ImmunoSpot Analyzer and CTL BioSpot software (Cellular Technology Ltd., Shaker Heights, OH, USA).

### Measurement of serum cytokines via multiplex Luminex bead assays

Frozen plasma at -80°C was thawed on ice, vortexed, and then centrifuged for 10 minutes to remove debris and aggregates. A total of 25 to 50 μl of serum were used for Luminex assays (Invitrogen) following the manufacturer's instruction. Ten different mouse cytokines were measured using the mouse cytokine 10-plex bead kit (Invitrogen). Briefly, filter plates (Millipore) were prewetted with 200 μl of wash solution (kit component), and 25 μl of beads were added per well. Serum samples were diluted, and a total volume of 50 μl/well was added (that is, 25 μl of sample serum plus 25 μl of assay diluent). Plates with beads were incubated for 2 hours at RT on an orbital shaker in the dark. At the end of the incubation, the plates were washed twice in buffer and 100 μl of secondary biotinylated antibody were added at a 1:10 dilution in biotin diluent provided with the kit. Plates were incubated at RT for 1 hour in the dark, then washed twice in buffer. Streptavidin in assay diluent was added at 100 μl/well, then incubated for 30 minutes at RT in the dark. Plates were washed three times with 100 μl of wash solution and agitated for 2 to 3 minutes at RT in the dark. Plates were run immediately on a Luminex xMAP 200 unit with data acquisition and analysis software (Invitrogen). All bead washing was performed using a vacuum manifold unit (Pall, Port Washington, NY, USA).

### Measurement of paw cytokines and pSTAT levels via multiplex Luminex bead assays

The method described in this subsection has been published elsewhere [25]. Briefly, the front (including carpus) and back (including tarsus) right paws were collected, frozen in dry ice-cooled isopentane, and stored at -80°C until use. The paws were cut to 2 mm × 2 mm segments and kept on dry ice in round-bottomed polypropylene tubes. Seven hundred microliters of tissue extraction buffer containing protease inhibitor cocktail (Calbiochem, San Diego, CA, USA) and Halt phosphatase inhibitor cocktail (Thermo Scientific) or Roche phosphatase inhibitor cocktail (Roche, Indianapolis, IN, USA) in tissue extraction reagent I (Invitrogen) were added to each sample. Samples were homogenized frozen using a PT 10/-35 Polytron Homogenizer (Brinkmann, Dallas, TX, USA). After homogenization, samples were centrifuged at 4°C and 2,000 × *g *for 10 minutes, and the supernatants were recentrifuged at 4°C at maximum speed, 14,000 × *g*, for 15 minutes. Supernatants were carefully removed to avoid picking up the top layer of lipids/adipose debris. The protein concentration was measured using a bicinchoninic acid protein assay (Pierce Biotechnology) and adjusted to 3 mg/ml. A minimum of 25 μl of this extraction was used in the above-described protocol using the mouse cytokine 10-plex bead kit (Invitrogen) or STAT1, 3, 5a/b Phospho 3-Plex Panel (Invitrogen).

### Compound quantification (Pharmacokinetics)

Plasma and protein extracted from paw and spleen samples were submitted for quantitative analysis to determine the respective compound concentrations. Blood samples were collected into heparinized tubes and placed on wet ice until being centrifuged (16,000 × *g *for 5 minutes) to separate the plasma. Supernatant was collected and stored at -20°C pending analysis. At the time of analysis, two volumes of cold acetonitrile containing an internal standard (alprenolol) were added to each sample, which were then vortexed and centrifuged. The supernatants were removed and placed into an autosampler vial, and the amount of compound present in the samples was analyzed by liquid chromatography/mass spectrometry. The concentration of compound in the samples was quantified against a mouse plasma standard curve made via serial dilution in a concentration range from 5 to 20,000 ng/ml. Samples containing concentrations more than 10% above the top of the standard curve were diluted 1:10 with acetonitrile. The limits of detection for plasma, paw, and spleen were <10 ng/ml.

### Statistical analysis

All ELISA or Luminex assays were analyzed using linear regression curves to determine the concentration of analyte following data acquisition. Mann-Whitney nonparametric one- or two-way analysis of variance (ANOVA) was used as for statistical testing as noted in the figure legends, depending on the experiment and the tested hypothesis. Bonferroni posttests were used to compare replicates in ANOVA tests. A *P *value < 0.05 was considered significant. For all studies, a minimum of 10 mice were included in each experimental group. The statistical software used was GraphPad Prism version 5.01 (GraphPad, La Jolla, CA, USA), and calculations were performed using Microsoft Excel software (Microsoft Corp., Redmond, WA, USA).

## Results

### *In vitro *and *in vivo *characterization of the JAK2 inhibitory activity of CEP-33779

**Figure 1:**
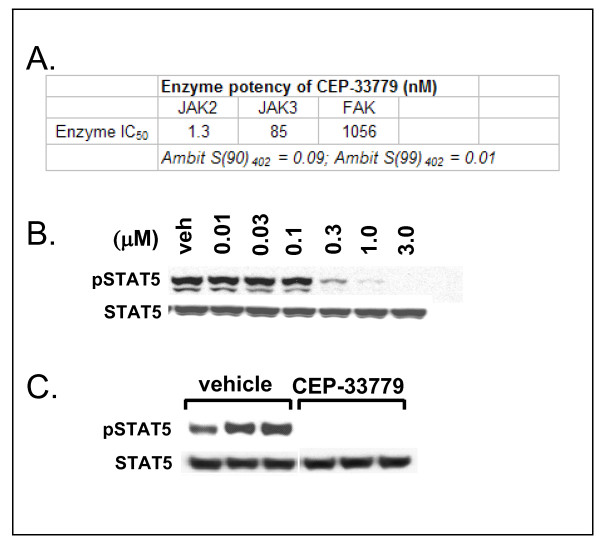
**Biochemical and cellular characterization of a selective Janus kinase 2 (JAK2) inhibitor, CEP-33779**. **(A) **Enzymatic inhibitory activities of CEP-33779 against JAK2 and family members. Inhibition of the kinase activity of each recombinant enzyme (JAK2 and JAK3) by CEP-33779 was evaluated using a plate-based, time-resolved fluorescence detection system. The effect in a cellular system was measured using irf-*bla *TF-1 cells (JAK2) and Ba/F3 JAK3 cells not tested for JAK1 and TYK2. Half maximal inhibitory concentration (IC_50_) values are reported as the averages ± SD of at least four independent determinations. FAK, focal adhesion kinase. **(B) ***In vitro *activity of CEP-33779 in HEL92 cells and potent JAK2 inhibition as determined by the inhibition of phosphorylation of downstream target signal transducer and activator of transcription 5 (pSTAT5), vehicle (veh). HEL92 cells were treated with increasing concentrations of CEP-33779 as indicated for 1 hour in serum-free media. Extracts were prepared in a Triton X-100-based lysis buffer, protein concentrations were determined, and equal amounts were resolved on sodium dodecyl sulfate polyacrylamide electrophoresis (SDS-PAGE) gels and blotted. STAT5 and pSTAT5 were analyzed using specific antibodies (see Materials and methods). Blots were scanned, and signals for each group were determined using GelPro as phosphor/total. Prism software was used to calculate the IC_50 _values. **(C) ***In vivo *pharmacodynamic (PD) in which mice were dosed orally (p.o.) with 55 mg/kg CEP-33779 before the removal of HEL92 tumor extracts (2 hours postinjection) and quantitation of STAT5 and pSTAT5 levels using Western blot analysis. Each lane represents an individual sample. Profiling studies for CEP-33779 were repeated for HEL92 cells and performed in several tumor lines both *in vitro *and *in vivo*, reconfirming JAK2 inhibition (data not shown).

CEP-33779 was synthesized and identified as a potent inhibitor of the target kinase in an assay of the isolated human enzyme (IC_50 _= 1.8 ± 0.6 nM) (Figure 1A). When evaluated against the other members of the JAK family, CEP-33779 demonstrated varying degrees of selectivity from >40-fold versus JAK1 to >800-fold against TYK2 (Figure 1A). The broad selectivity of CEP-33779 with respect to inhibition of other members of the kinome was assessed using binding assays for a panel of 402 kinases [26]. At a test concentration of 1 μM, CEP-33779 was highly selective, with 9% of the panel inhibited by >90% and only 1% inhibited by >99% (S(90)_402 _= 0.09 and S(99)_402 _= 0.01, respectively), using the selectivity score defined by Karaman *et al. *[27] (see also Figure 1A). In a cellular system, CEP-33779 was shown to inhibit JAK2 in irf-*bla *TF-1 cells utilizing the GeneBLAzer reporter assay (Invitrogen) (data not shown) and in HEL92 cells by measuring the phosphorylation of STAT5 (Figure 1B). Activity against pSTAT3 was also observed (data not shown); however, STAT3 can be activated via both the epidermal growth factor receptor (EGFR) and Src kinase signaling pathways [28,29]. The lack of activity against either of these kinases in the profiling panel suggests that the effects on pSTAT3 were mediated by inhibition of JAK2. The activity of CEP-33779 was also demonstrated in an *in vivo *PD assay using HEL92 cells (Figure 1C). The *in vitro *and *in vivo *profiles of CEP-33779 were consistent with its identification as a potent and selective inhibitor of JAK2.

### JAK2 inhibition suppresses CAIA and inhibits local cytokine responses

**Figure 2:**
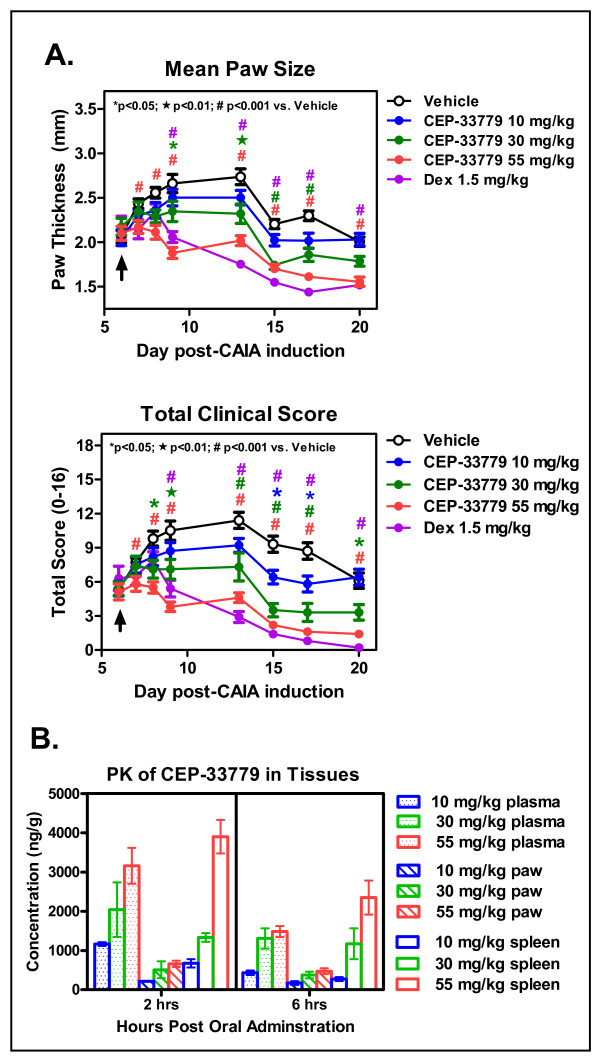
**JAK2 blockade ameliorates collagen antibody-induced arthritis (CAIA) paw inflammation**. Female DBA/1 mice were injected intravenously (i.v.) with purified anti-collagen type II (anti-CII) antibodies, and then 25 μg of lipopolysaccharide (LPS) was administered intraperitoneally (i.p.) 3 days later to induce arthritis by day 5. Mice that met the arthritis criteria score of 1 per paw were entered into the study. CEP-33779 was administered p.o. twice daily (b.i.d.) throughout the experiment, 1.5 mg/kg dexamethasone (Dex) was administered i.p. three times weekly and vehicle (PEG400 + 1% dimethyl sulfoxide (DMSO)) was administered p.o. **(A) **Mean paw size (individual paws measured for thickness) measured over time (top) and total clinical score from each paw were recorded (bottom). *P < 0.05; black star, *P *< 0.01; #*P *< 0.001. Statistical significance was measured by using two-way analysis of variance (ANOVA), *n *≥ 10 mice per group. **(B) **Pharmacokinetics (PK) of CEP-33779 at 55 mg/kg, 30 mg/kg and 10 mg/kg, p.o., once daily (s.i.d.), in the plasma, paw and spleen of treated mice, with statistical significance not determined; *n *= 4 mice per group tested. All graphs show means ± SEM. Study size shows *n *≥ 10 mice per group and *n *≥ 5 mice for individual assays. Black arrows in parts A and B indicate treatment start.

Anti-CII antibody was delivered to mice i.v., and oral administration of compound was initiated upon individual mice meeting minimum score requirements and being entered into dosage groups. CEP-33779 was administered at 10, 30, or 55 mg/kg p.o., b.i.d., for a total of 2 weeks. Vehicle groups were treated with PEG400 in 1% DMSO. Dex was used as a glucocorticoid standard of care reference and was given i.p. in saline solution alone three times weekly (every 48 hours) at 1.5 mg/kg total body mass. This high dose of Dex, which was chosen to define a fully treated disease index, is within the range (0.3 to 1.5 mg/kg) employed by several groups who have used inflammation models [30-32]. Individual paws were scored and measured using a standard electric caliper, and the measurements were averaged to produce the graphed results. Dose-dependent responses were evident by the reduction in mean paw thickness or size over time with treatment (1.3-fold drop at day 13 for 55 mg/kg dose compared to vehicle) (Figure 2A, top). This correlated well with a decrease in total clinical score peaking at the highest dose used, 55 mg/kg (2.4-fold reduction at day 13 for 55 mg/kg dose compared to vehicle) (Figure 2A, bottom). The efficacy observed at the 55 mg/kg dose of CEP-33779 paralleled that observed with Dex (for days 13 to 20) (Figure 2A). For all animals treated, total body mass was not significantly different from that of vehicle-treated animals (Additional file 1). In previous experiments, nondiseased animals showed similar weight gain compared to their vehicle-treated counterparts (data not shown). Analysis of CEP-33779 in plasma, paw, and spleen at 2 and 6 hours after oral administration on day 20 demonstrated dose-related increases in the levels of compound (Figure 2B). While compound levels in the target tissue, the paw, were relatively low, they were relatively constant between the two time points (average 659.0 ng/g at 2 hours for 55 mg/kg versus 472.0 ng/g at 6 hours for 55 mg/kg) (Figure 2C).

To investigate the effect of CEP-33779 on levels of pSTAT and cytokines in the paws of treated mice, a method was developed whereby synovial fluid and cellular joint extracts could be obtained from the paws and ankles of diseased animals for analysis using multiplex bead arrays [25]. On the basis of the activity of CEP-33779 as a JAK2 inhibitor, pSTAT5, pSTAT3, and possibly pSTAT1 were hypothesized to be reduced in the paws of treated mice as previously suggested on the basis of *in vitro *and *in vivo *experiments (Figures 1A and 1B and data not shown). Using multiplex bead kits, it was determined that measurement of pSTAT was similar and more quantitative than using Western blot analysis alone. Consequently, multiplex analysis was used as the primary method of measuring pSTAT for all further studies reported here [25].

**Figure 3:**
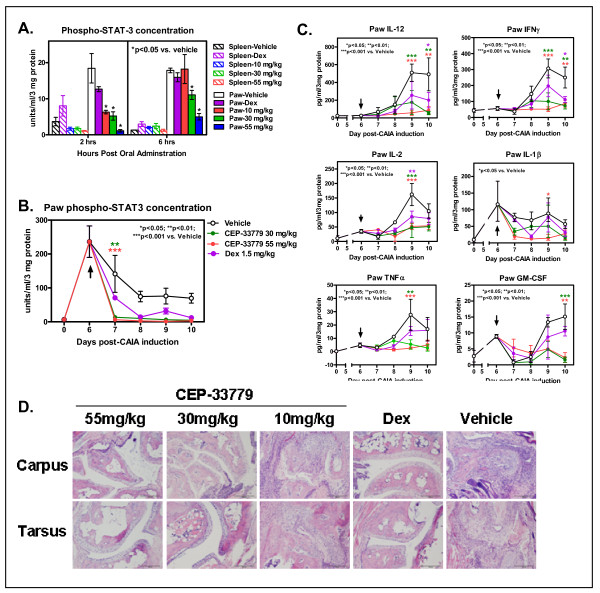
**Disease treatment reflected in local paw environment supports the use of a selective JAK2 inhibitor for suppressing joint inflammation**. DBA/1 female mice were injected i.v. with purified anti-CII antibodies, then administered 25 μg of LPS i.p. 3 days later to induce arthritis by day 5. Mice that met the arthritis criteria score of 1 per paw were entered into the study. CEP-33779 was administered p.o., b.i.d., throughout the experiment, Dex was administered at 1.5 mg/kg three times weekly i.p., and vehicle (PEG400 + 1% DMSO) was administered p.o. **(A) **Spleen and paw phospho-STAT3 concentrations were determined by using Luminex bead kits, with the concentrations shown along the *y*-axis in units per milliliter per 3 mg of total protein as determined by using a bovine serum albumin (BSA) assays. Tissues were removed 2 and 6 hours after administration of CEP-33779 p.o. at 55 mg/kg. Graph shows means ± SEM. *P < 0.05 by two-way ANOVA. **(B) **Paw phospho-STAT3 expression in CAIA mice over time with CEP-33779 treatment b.i.d. as measured using a Luminex kit. Black arrow indicates treatment start. **(C) **Paw cytokines were measured as described elsewhere [25]. Mouse 10-plex Luminex kits were used to measure paw cytokines. Cytokines shown include interleukin (IL)-12, interferon γ (IFNγ), IL-2, IL-1β, tumor necrosis factor α (TNFα) and granulocyte macrophage colony-stimulating factor (GM-CSF). Black arrows indicate treatment start. **(D) **Hematoxylin and eosin-stained, paraffin wax-embedded sections from treated mice show infiltrates and damaged joints. Representative (pathologist-selected) images are shown at × 10 original magnification. Tissues were also stained with Safranin O for matrix degeneration (data not shown). Tissues from five representative mice from each group were sent for histological analysis and graded according to an accepted scoring method (see Materials and methods). All graphs show means ± SEM, and all statistics were calculated using Prism software and two-way ANOVA analysis. *P < 0.05. **P < 0.01. ***P < 0.001. Study size shows *n *≥ 10 mice per group and *n *≥ 5 mice for individual assays.

Local paw pSTAT5 levels in diseased animals were similar to those in mice treated with vehicle alone ([25] and data not shown). However, pSTAT3 dominated as the most highly upregulated STAT molecule in diseased compared to nondiseased animals and was significantly reduced in total concentration upon JAK2 inhibition (Figure 3A and data not shown). pSTAT1 was barely detectable in all samples tested (data not shown). pSTAT3 levels were nearly abolished in the paw 2 hours after CEP-33779 treatment (Figure 3A, left) and only slowly began to recover at 6 hours after CEP-33779 treatment (Figure 3A, right). The expression of total paw pSTAT3 decreased during the course of disease when treated with CEP-33779 as compared to vehicle control mice (Figure 3B). Levels of pSTAT3 were elevated in CAIA mice because of the requirement to prime mice with 25 μg of LPS on day 3 after antibody transfer; however, disease maintenance was evident in the sustained paw inflammation and pSTAT3 activity over time in these animals (Figure 3B). Within 24 hours after CEP-33779 b.i.d. treatment, the reduction in pSTAT3 level was significantly greater than that of Dex as compared to vehicle alone (Figure 3B, days 6 and 7; *P *< 0.01 for 30 mg/kg and *P *< 0.001 for 55 mg/kg as compared to vehicle). This reduction of pSTAT3 was reached by Dex treatment 48 hours after treatment initiation (Figure 3B, days 6-8). CEP-33779 reduced pSTAT3 expression to the lower limits of the assay throughout the remainder of the studies (Figure 3B).

Reduced paw swelling accompanied by a reduction in pSTAT3 expression strongly suggested that, at these levels, a decrease in the expression of several cytokines controlled by STAT3 should be observed. In a repeat of the previous experiment using the 55 mg/kg and 100 mg/kg doses, paw extracts were analyzed for the expression level of several proinflammatory cytokines. Both doses of CEP-33779 significantly reduced the level of several of the cytokines implicated in the pathogenesis of RA (for example, IL-1β, TNFα, and IL-6) (Figure 3C and Additional file 2, Figure S2C) [33]. Dose-dependent reductions were observed for all cytokines tested in paw extracts, especially for IL-12, IL-2, GM-CSF and IL-4 (Figure 3C, *P *< 0.01 compared to vehicle; Additional file 2, Figure S2A). For several of these cytokines, CEP-33779 significantly decreased cytokine expression below that of high-dose Dex alone (Figure 3C, *P *< 0.05 compared to vehicle), and the 100 mg/kg dose lowered and sustained cytokine suppression for the remainder of the study (Figure 3C, IL-1β, IL-6, TNFα, IL-12; *P *< 0.01 compared to vehicle). Histological analyses of decalcified front and hind limbs showed that treatment with CEP-33779 reduced several clinical features associated with progressive RA (Figure 3D). Independent pathologist scoring and image collection allowed us to determine that treatment with CEP-33779 could significantly decrease several parameters, including matrix erosion, subchondral osteolysis, osteoproliferation, synovial proliferation, pannus formation, and degree of inflammation, as compared to vehicle controls (Table 1). Dose-dependent responses upon JAK2 inhibitor treatment resulted in reduced bone degradation, reduced tissue destruction, and reduced osteoarthritis as evidenced by the histopathology images (Figure 3D).

**Table 1:** CAIA model joint inflammation histopathology scores^a^

Group	Proteoglycan loss	Matrix erosion	Subchondral osteolysis	Osteoproliferation	Synovial proliferation	Pannus formation	Inflammation degree
Vehicle	1.55 ± 0.07	3.12 ± 0.71	2.50 ± 1.00	1.65 ± 0.47	2.00 ± 0.81	1.12 ± 0.85	1.75 ± 0.95
CEP-33779 10 mg/kg	1.39 ± 0.38	3.40 ± 2.07	2.30 ± 1.30	1.70 ± 1.09	2.40 ± 1.34	1.30 ± 0.97	1.60 ± 1.14
CEP-33779 30 mg/kg	1.12 ± 0.88	0.75 ± 0.95	0.62 ± 0.47*	0.87 ± 0.62	0.25 ± 0.50*	0.00 ± 0.00	0.12 ± 0.25*
CEP-33779 55 mg/kg	2.05 ± 0.07	0.70 ± 0.44*	0.60 ± 0.41**	0.30 ± 0.72*	0.30 ± 0.44*	0.00 ± 0.00*	0.20 ± 0.27*
Dex 1.5 mg/kg	0.98 ± 0.44	1.00 ± 0.79	0.50 ± 0.35**	0.70 ± 0.44	0.40 ± 0.54*	0.10 ± 0.22	0.30 ± 0.44*
Compared to vehicle	*P < 0.05	**P < 0.01	***P < 0.001				

^a^CAIA, collagen-antibody induced arthritis; Dex, dexamethasone. Hematoxylin and eosin- and Safranin O-stained, paraffin wax-embedded carpus and tarsus sections were scored by a board-certified independent pathologist for the criteria described in Materials and methods. Values are means ± SD for the tarsus only. *P *values show significance as compared to vehicle group; *n *= 5 mice per group. Statistical test used was two-way analysis of variance (ANOVA). *P < 0.05. **P < 0.01. ***P < 0.001.

### Inhibiting JAK2 slows the progression of chronic CIA and reduces the generation of disease-promoting Th1 cells

**Figure 4:**
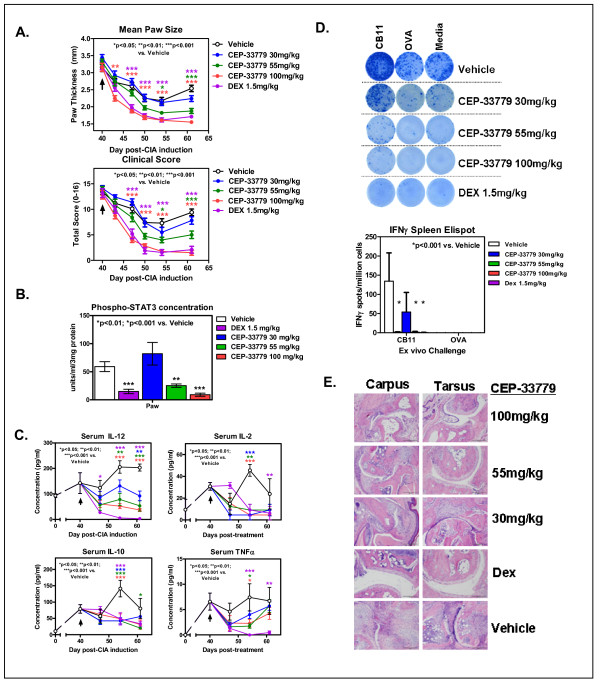
**JAK2 blockade reduces several disease correlates in a model of chronic degenerative arthritis**. Female DBA/1 mice were injected with purified CII in Complete Freund's Adjuvant (CFA) i.d., then boosted with CII in Incomplete Freund's Adjuvant (IFA) s.c. on day 21 followed by a day 28 LPS injection i.p. to induce collagen-induced arthritis (CIA). Mice that scored 1 or better for each paw were considered arthritic and entered into the study. Treatments started after 7 to 10 days of full arthritis. CEP-33779 was administered p.o., b.i.d., throughout the remainder of the experiment. Dex was administered i.p. at 1.5 mg/kg three times weekly, and vehicle was PEG400 + 1% DMSO p.o., b.i.d. **(A) **Mean paw size (individual paws measured) and clinical scores for each mouse are graphed. **(B) **Phospho-STAT3 expression in the paws of treated CIA mice. **(C) **Serum cytokines (IL-12, IL-2, IL-10 and TNFα) from treated CIA mice. **(D) **Representative ELISPOT images showing IFNγ enzyme-linked immunosorbent spot (ELISPOT) assay results from stimulated splenocytes from treated mice challenged with CII fragment (CB11), chicken ovalbumin (OVA) or media alone. Graph was constructed using data from five mice tested from each group. Spots were counted using a CTL ImmunoSpot ELISPOT scanner and spot-counting software. **(E) **Hematoxylin and eosin-stained, paraffin wax-embedded sections from treated CIA mice showing joint infiltrates and bone resorption. Representative (pathologist-selected) images are shown at × 10 original magnification. Safranin O-stain sections are not shown but were used for scoring. All graphs show means ± SEM, and all statistics were calculated using Prism software and two-way ANOVA. *P < 0.05. **P < 0.01. ***P < 0.001. Study size shows *n *≥ 10 mice per group and *n *≥ 5 mice for individual assays. Black arrows in parts A and C indicate treatment start.

To test JAK2 inhibitors in a more physiologic and progressive disease model of RA (that is, the CIA model), arthritic mice that previously had been sensitized against self-CII and exhibited the phenotype of natural RA were treated with CEP-33779. Laboratory studies demonstrated that this CIA model exhibited arthritic flares as previously reported in the literature (data not shown) [18]. Early model optimization studies consistently generated robust CIA phenotypes that were absent from control-treated, nondiseased animals (data not shown). CIA animals were treated with CEP-33779 at 30, 55, and 100 mg/kg doses b.i.d, p.o. Vehicle and the standard-of-care reference drug, Dex, at 1.5 mg/kg, were administered as described above. There was a clear reduction in the mean paw size (individual values for paws collected but not shown), along with a reduction in clinical score, in a dose-dependent manner observed in treated mice (Figure 4A, top and bottom). The most striking observation was the nearly identical suppression of paw swelling in mice treated with 100 mg/kg CEP-33779 versus those treated with high-dose Dex (Figure 4A, red and violet lines). Also, at least with regard to the objective parameter of mean paw size, there was a drop in the swelling of the 100 mg/kg treated group preceding that of the Dex-treated group (Figure 4A). Again, differences in the mechanism of action may account for this result. As expected, pSTAT3 was also significantly reduced in mice treated with CEP-33779 at 55 mg/kg (Figure 4B, *P *< 0.01) and 100 mg/kg (Figure 4B, *P *< 0.001). For all three CEP-33779 doses, reductions in local paw pSTAT3 expression directly corresponded to decreases in several serum and paw cytokines involved in the disease progression of arthritis (Figure 4C, IL-12, IL-2, IL-10, and TNFα; *P *< 0.05) (Additional file 2, Figure S2A; IL-4, *P *< 0.001) and spleen IL-12 (Additional file 2, Figure S2B; *P *< 0.05). A reduction in paw IL-6 (Additional file 2, Figure S2C; *P *< 0.05) with CEP-33779 treatment was also observed. However, this cytokine was difficult to monitor because of the IL-6-driven flares causing spikes in the vehicle groups (Additional file 2, Figure S2C). Body mass did not significantly change for any group tested; however, mice treated with CEP-33779 appeared healthier and retained greater body mass longer than those in the control groups (unpublished observations, KL Stump) (Additional file 3).

The dose response of CEP-33779 treatment was most clearly observed when IL-12, the main precursor and driver of pathogenic Th1 and Th17 cells [34,35], was measured (Figure 4C). High-dose Dex was effective in suppressing endogenous IL-12 expression in the sera of arthritic mice, but all three doses of CEP-33779 were able to significantly decrease the level of IL-12 compared to that observed with Dex (Figure 4C). Since IL-12 is the differentiating cytokine for Th1 cells (via T-bet expression) and anti-CII Th1 cells are the main drivers of paw inflammation in the CIA model, it was hypothesized that if CEP-33779 can suppress circulating IL-12, then the total frequency of CII-specific T cells should be reduced upon treatment. With the knowledge that the same Th1 cells found locally in the paw must have circulated throughout the animal and entered secondary lymphoid organs, whole splenocytes from treated CIA mice were restimulated with the purified CII fragment (CB11) containing immunodominant epitopes *ex vivo *in IFNγ T-cell ELISPOT assays. As expected, there was a significant reduction in the frequency of CB11-specific, IFNγ-secreting T cells when treated with either Dex or CEP-33779 at 55 mg/kg or 100 mg/kg b.i.d, p.o., for 4 weeks (Figure 4D; *P *< 0.001). Representative ELISPOT images demonstrate that higher doses of CEP-33779 result in a greater reduction in the frequency of Th1-like cell responses (Figure 4D). However, a reduction in anti-CII IgG serum titers in CEP-33779-treated mice was not observed (Additional file 4), suggesting that the root of the problem, autoreactive B cells, still has to be addressed [36]. Anti-CII antibodies were not present in nonarthritic mice, and anti-CI antibodies were tested but never found in this experiment or in the CIA model (data not shown). Disease treatment using CEP-33779 led to less paw inflammation and bone deterioration as demonstrated by histological analysis (Figure 4E) and independent, blinded pathologist scores (Table 2). In the CIA model, the tarsus tends to become more inflamed than the carpus, and the opposite is observed in the CAIA model (unpublished data, KL Stump). Both 100 mg/kg and 55 mg/kg CEP-33779 doses provided protection in the tarsus, but 100 mg/kg CEP-33779 provided the best protection for both regions (Figure 4E). However, significance was observed only for the degree of inflammation in the CIA model for both the 100 mg/kg and 55 mg/kg CEP-33779 doses (Table 2) (*P *< 0.01 for 100 mg/kg and *P *< 0.05 for 55 mg/kg compared to vehicle controls).

**Table 2:** CIA model joint inflammation histopathology scores^a^

Group	Proteoglycan loss	Matrix erosion	Subchondral osteolysis	Osteoproliferation	Synovial proliferation	Pannus formation	Inflammation degree
Vehicle	3.00 ± 0.00	3.67 ± 1.52	2.50 ± 0.50	2.66 ± 0.57	2.66 ± 0.57	1.66 ± 0.57	2.83 ± 0.28
Compound A 30 mg/kg	2.50 ± 0.57	4.62 ± 0.75	2.75 ± 0.50	2.50 ± 0.40	2.75 ± 0.50	2.37 ± 0.94	2.12 ± 0.25
Compound A 55 mg/kg	1.83 ± 0.76	2.83 ± 1.60	1.33 ± 1.04	1.83 ± 1.15	1.00 ± 0.86	0.83 ± 0.76	0.66 ± 0.57*
Compound A 100 mg/kg	1.83 ± 0.28	2.33 ± 1.52	1.50 ± 0.50	1.66 ± 0.57	1.50 ± 0.86	0.66 ± 1.15	1.33 ± 0.57**
Dex 1.5 mg/kg	2.25 ± 0.95	3.12 ± 2.09	1.37 ± 0.94	1.50 ± 1.08	1.25 ± 0.86	0.75 ± 0.64	1.00 ± 0.81**
Compared to vehicle	*P < 0.05	**P < 0.01	***P < 0.001				

^a^CIA, collagen-induced arthritis. Hematoxylin and eosin- and Safranin O-stained, paraffin wax-embedded carpus and tarsus sections were scored by a board-certified independent pathologist for the criteria described in Materials and methods. Values are means ± SD for the tarsus only. *P *values show significance compared to vehicle group; *n *= 5 mice per group. Statistical test used was two-way ANOVA. *P < 0.05. **P < 0.01. ***P < 0.001.

### Inhibition of cellular immunity with JAK2 inhibition using an air pouch inflammation model

**Figure 5:**
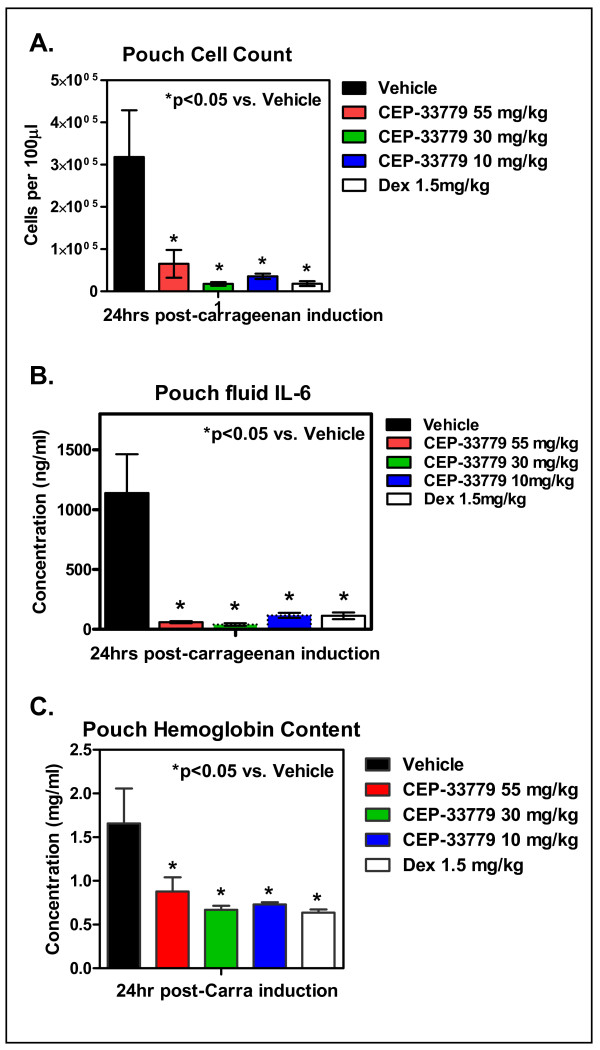
**Suppression of carrageenan-induced inflammation by CEP-33779**. Balb/c mice were prepared for air pouch initiation (see Materials and methods) using sterile air. Carrageenan at 1% in saline solution was injected directly into the pouch, followed immediately by CEP-33779 or Dex treatment 1 hour later. One hundred microliters of air pouch fluid were removed 24 hours after compound administration. **(A) **Total cell count using 7-aminoactinomycin D exclusion via flow cytometeric analysis and BD TruCount beads. **(B) **Fluid supernatant IL-6 concentration as determined by Luminex cytokine analysis. **(C) **Hemoglobin content was assessed by the Drabkin's assay. All graphs show means ± SEM. *P < 0.05 with two-way ANOVA used as the statistical test; *n *= 5 mice per group tested.

To test the impact of CEP-33779 inhibition of JAK2 at the cellular level, the mouse APM was utilized. Air-filled subcutaneous pouches were maintained under sterile conditions and injected with 1% carrageenan simultaneously with CEP-33779 at 10, 30, and 55 mg/kg. Saline solution alone (vehicle) or Dex at 1.5 mg/kg was used as the control. CEP-33779 JAK2 inhibition significantly decreased cellular accumulation in the air pouch (Figure 5A; *P *< 0.05 compared to vehicle). CEP-33779 JAK2 inhibition could also inhibit the expression of IL-6 to less than that of Dex (Figure 5B; *P *< 0.05 compared to vehicle). In addition, angiogenesis was affected as measured using the indirect angiogenesis assay (Drabkin's assay) to measure the total hemoglobin content of the pouch contents (Figure 5C; *P *< 0.05 compared to vehicle). APM treatment with CEP-33779 provided similar results when administered at 1 to 0.1 mg/kg (data not shown). These data suggest that CEP-33779 inhibition of JAK2 directly affects cellular immune function, and thus the mechanism of action in the RA models must be mediated at the level of leukocyte flux and cytokine release and/or response.

## Discussion

Here we report the efficacy profile of CEP-33779, a highly selective, orally active, small-molecule inhibitor of JAK2 that we evaluated in two mouse models of RA. PD inhibition of JAK2 reduced mean paw edema and clinical scores in both the CIA and CAIA models of arthritis. Reduction in paw and serum cytokines correlated with reduced spleen CII-specific Th1 cell frequencies as measured by *ex vivo *IFNγ ELISPOT assays. Both models demonstrated histological evidence of disease amelioration upon treatment and reduced paw pSTAT3 levels. This study demonstrates the utility of using a potent and highly selective, orally bioavailable JAK2 inhibitor for the treatment of RA.

The CIA model is dependent on both T- and B-cell responses and, as in human RA, depends on the action of IL-6, demonstrating that CEP-33779 could modulate key immune cytokines involved in human disease [15,37,38]. Unlike the CIA model, the CAIA model has been shown not to be entirely dependent on IL-6, as arthritis can still be induced in mice deficient in this cytokine [39]. However, surprisingly, robust activity of CEP-33779 was observed in the CAIA model, sometimes exceeding that of the standard-of-care agent Dex. The use of tocilizumab demonstrates how targeting cytokines can directly affect disease progression without overt immunosuppression [8]. Other small molecules such as INCB028050 have also been reported to show a similar phenotype, that is, inhibition of IL-6 and disease without immunosuppression [14]. It is possible that CEP-33779 works similarly to INCB028050, as both can inhibit immune cellular flux and cytokine release in acute inflammation mouse models (that is, delayed hypersensitivity for INCB028050 and the APM model for CEP-33779) (Figure 5).

JAK2 is responsible for mediating signaling through several receptors, including those highly implicated in playing an essential role in autoimmunity and inflammation, such as the gp130 receptor family members IL-6R and IL-12Rβ and IFNγR2 [40]. Blockade of JAK2 associated with these major cytokine receptors may affect the subsequent expression or production of downstream cytokines during arthritis and may be responsible for some of the results shown here. For example, reduction of cytokines IL-12, IL-2, and TNFα may switch the local milieu from a strong Th1/Th17 phenotype to a more immunoregulatory Th2/regulatory T cell phenotype and lead to disease symptom reduction [41].

Interactions of JAK2 with various immune-related signaling molecules will determine how inhibition of this kinase may affect other immune cell types and thus the treatment of other autoinflammatory diseases. Downstream JAK2 can interact with STAT5a/b, STAT3, STAT4, and STAT1 [42]. However, only STAT5a/b are directly activated by JAK2; STAT4 can be activated via TYK2, STAT1 can be activated via JAK1, and STAT3 can be activated via the EGFR pathway and JAK1 and Src kinases [28,29]. In addition, several kinases, including Tec, Vav1, Fyn, Yes1, and protein tyrosine kinase 2, also directly interact with JAK2 [43-47]. These kinases are all involved at some level in the regulation and activation of immune subsets, especially those of T and B lymphocytes. Inhibiting JAK2 may have implications other than just direct inhibition of cytokine signaling. Downstream inhibition of T- and B-cell activation may also result from JAK2 inhibition, expanding the possibilities of using a potent inhibitor of this kinase in alternate indications.

Suppressors of the JAK-STAT signaling pathway, suppressors of cytokine signaling (SOCS), also interact directly with JAK2 (for example, SOCS3) [42,48]. SOCS proteins act as a negative feedback loop for the JAK-STAT pathway [49]. Interestingly, the expression of SOCS is altered in RA patients; levels of SOCS1 and SOCS3 were shown to be increased in peripheral blood mononuclear cells (PBMCs) of RA patients [50]. SOCS1 was upregulated in peripheral blood T cells associated with SOCS3 expression in PBMCs in patients with RA [50]. SOCS1 was also showed to be upregulated in the synovial membranes of patients with RA compared to patients with osteoarthritis. This suggests that deregulation of JAK2 signaling via effects on SOCS can contribute to RA development and may predict a patient's susceptibility to disease development.

## Conclusions

This report demonstrates the use of a highly selective and potent JAK2 inhibitor, CEP-33779, for the treatment of RA using two different mouse models of RA. Inhibition of disease-promoting cytokine pathways by CEP-33779 nearly rivaled that of standard-of-care treatments, strongly suggesting the use of this inhibitor in other IL-6-dependent diseases, such as Crohn's disease and ulcerative colitis. CEP-33779 may also have utility in other diseases dependent on the same pathways such as systemic lupus erythematosus, inflammatory bowel disease, psoriasis, and various cancers, including colon, prostate, and pancreatic cancer. These results warrant further preclinical evaluation of selective JAK2 inhibitors such as CEP-33779.

## Abbreviations

CII: type II collagen; CAIA: collagen antibody-induced arthritis; CIA: collagen-induced arthritis; RA: rheumatoid arthritis; RT: room temperature.

## Competing interests

All authors of this paper are employees of Cephalon, Inc. The authors declare that they have no competing interests.

## Authors' contributions

KLS was responsible for the generation of the CAIA and CIA model systems. LDL was responsible for several of the immunoassay readouts, including anti-CII ELISA and serum cytokine panels, and for developing the paw extraction protocol for pSTAT and cytokine quantitation. MMS supervised, and was the lead investigator for, the use of CEP-33779 in mouse models of RA; prepared the manuscript; and performed all the T-cell ELISPOT assays and flow cytometry. BAR supervised the *in vitro *and *in vivo *use of CEP-33779 for multiple therapeutic indications, including RA, and edited the manuscript. MSA performed JAK enzyme inhibition studies. TSA supervised and analyzed data gathered from JAK enzyme and cell inhibition studies. MAA supervised biochemical and pharmacokinetic studies and edited the manuscript. PD supervised, and CS was involved with, the initial characterization of CEP-33779 as a JAK2 inhibitor in several cell lines and tumor models. BDD, DEG, and BJD were all involved with the design and synthesis of selective JAK2 inhibitors from among which CEP-33779 was selected as a preclinical candidate. All authors read and approved the final version of the manuscript.

## Supplementary Material

Additional file 1 **Total body mass of CEP-33779-treated collagen antibody-induced arthritis (CAIA) model mice over time**. Graph shows means ± SEM of total body mass of CAIA mice. Female DBA/1 mice were treated with CEP-33779 orally twice daily (b.i.d.) and dexamethasone (Dex) at 1.5 mg/kg three times weekly, and vehicle PEG400 + 1% dimethyl sulfoxide (DMSO) was administered orally (p.o.) b.i.d. Study size shows *n *≥ 10 mice per group and *n *≥ 5 mice for individual assays. Black arrow indicates treatment start. Click here for file

Additional file 2 **Reduction of serum interleukin (IL)-6 and IL-4 along with spleen IL-12 in collagen type II (CII)-induced arthritis (CIA) mice treated with CEP-33779**. Female DBA/1 mice were injected with purified CII in Complete Freund's Adjuvant (CFA) intradermally (i.d.), then boosted with CII in Incomplete Freund's Adjuvant (IFA) administered subcutaneously (s.c.) on day 21 followed by a day 28 lipopolysaccharide (LPS) injection intraperitoneally (i.p.) to induce CIA. Mice that scored 1 or better for each paw were considered arthritic and entered into the study. Treatments started after several days of full arthritis. CEP-33779 was administered p.o., b.i.d., throughout the remainder of the experiment. Dex was injected i.p. at 1.5 mg/kg three times weekly, and vehicle PEG400 + 1% DMSO was administered p.o., b.i.d. **(A) **Paw IL-4 concentration from treated CIA mice 4 hours post-oral dosing. **(B) **Cytokine IL-12 was measured using Luminex kits (Invitrogen) for paws and spleens from treated mice 4 hours after p.o. dosing. **(C) **Paw IL-6 concentration from treated CIA mice 4 hours after p.o. oral dosing. Values are means ± SD for the tarsus only. *P *values show significance compared to the vehicle group; *n *= 5 mice per group scored for both CAIA and CIA groups. The statistical test used was two-way analysis of variance (ANOVA). *P < 0.05. **P < 0.01. ***P < 0.001. Study size shows *n *≥ 10 mice per group and *n *≥ 5 mice for individual assays. Black arrow indicates treatment start. Click here for file

Additional file 3 **Total body mass of CEP33779-treated CIA model mice**. Graph shows means ± SEM of total body mass of CIA mice. Female DBA/1 mice were treated with CEP-33779 p.o., b.i.d., Dex at 1.5 mg/kg three times weekly, and vehicle PEG400 + 1% DMSO p.o., b.i.d. Study size shows *n *≥ 10 mice per group and *n *≥ 5 mice for individual assays. Black arrow indicates treatment start. Click here for file

Additional file 4 **No change in serum anti-CII autoantibodies in CIA mice treated with CEP-33779 over time**. Female DBA/1 mice were injected with purified CII in CFA i.d., then boosted with CII in IFA s.c. on day 21 followed by a day 28 LPS injection i.p. to induce CIA. Mice that scored 1 or better for each paw were considered arthritic and entered into the study. Treatments started after several days of full arthritis. CEP-33779 was administered p.o., b.i.d., throughout the remainder of the experiment. Dex was administered i.p. at 1.5 mg/kg three times weekly, and vehicle PEG400 + 1% DMSO was administered p.o., b.i.d. Serum testing using an anti-CII autoantibody enzyme-linked immunosorbent assay (ELISA) is described in Materials and methods and elsewhere [18]. No statistical significance was observed for any group compared to vehicle using two-way ANOVA. Serum dilution used was a five fold dilution for all samples, with the diluted sample shown. Graph shows means ± SEM, *n *≥ 10 mice per group tested, with serum collected at weekly intervals and stored at -80°C until tested. Study size shows *n *≥ 10 mice per group and *n *≥ 5 mice for individual assays. Black arrow indicates treatment start. Click here for file
