# Supplementary figures and images for: A highly selective, orally active inhibitor of Janus kinase 2, CEP-33779, ablates disease in two mouse models of rheumatoid arthritis
Source: Arthritis Res Ther. 2011 Apr 21;13(2):R68. doi: 10.1186/ar3329 (PMC3132063; doi:10.1186/ar3329)

### Total Body Mass

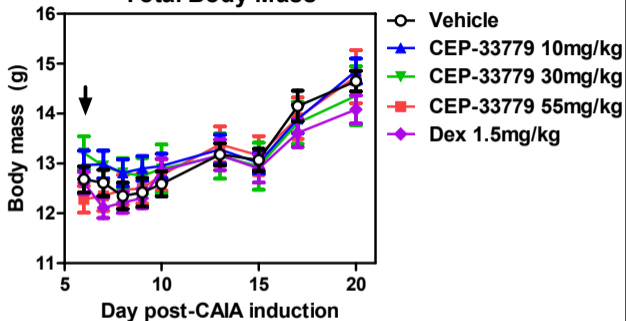

**Figure S1.0**

Supplement: Additional file 1 — Total body mass of CEP-33779-treated collagen antibody-induced arthritis (CAIA) model mice over time. Graph shows means ± SEM of total body mass of CAIA mice. Female DBA/1 mice were treated with CEP-33779 orally twice daily (b.i.d.) and dexamethasone (Dex) at 1.5 mg/kg three times weekly, and vehicle PEG400 + 1% dimethyl sulfoxide (DMSO) was administered orally (p.o.) b.i.d. Study size shows n ≥ 10 mice per group and n ≥ 5 mice for individual assays. Black arrow indicates treatment start. [file ar3329-S1.PDF]

**A.****Paw IL-4**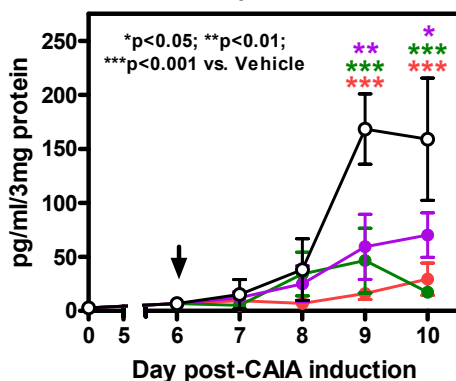**B.****IL-12 Concentration**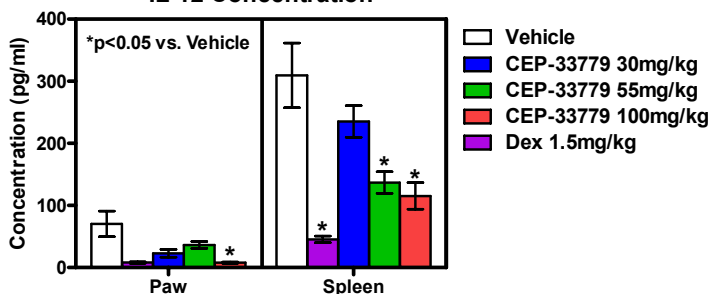**C.****Paw IL-6**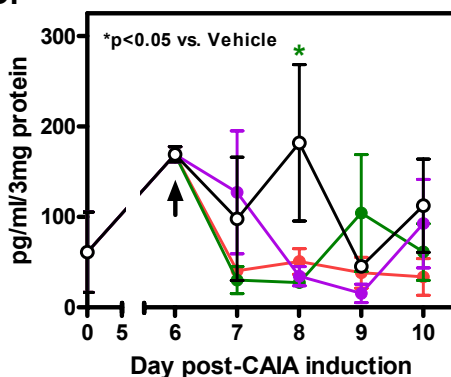**Figure S2.0.**

Supplement: Additional file 2 — Reduction of serum interleukin (IL)-6 and IL-4 along with spleen IL-12 in collagen type II (CII)-induced arthritis (CIA) mice treated with CEP-33779. Female DBA/1 mice were injected with purified CII in Complete Freund's Adjuvant (CFA) intradermally (i.d.), then boosted with CII in Incomplete Freund's Adjuvant (IFA) administered subcutaneously (s.c.) on day 21 followed by a day 28 lipopolysaccharide (LPS) injection intraperitoneally (i.p.) to induce CIA. Mice that scored 1 or better for each paw were considered arthritic and entered into the study. Treatments started after several days of full arthritis. CEP-33779 was administered p.o., b.i.d., throughout the remainder of the experiment. Dex was injected i.p. at 1.5 mg/kg three times weekly, and vehicle PEG400 + 1% DMSO was administered p.o., b.i.d. (A) Paw IL-4 concentration from treated CIA mice 4 hours post-oral dosing. (B) Cytokine IL-12 was measured using Luminex kits (Invitrogen) for paws and spleens from treated mice 4 hours after p.o. dosing. (C) Paw IL-6 concentration from treated CIA mice 4 hours after p.o. oral dosing. Values are means ± SD for the tarsus only. P values show significance compared to the vehicle group; n = 5 mice per group scored for both CAIA and CIA groups. The statistical test used was two-way analysis of variance (ANOVA). *P < 0.05. **P < 0.01. ***P < 0.001. Study size shows n ≥ 10 mice per group and n ≥ 5 mice for individual assays. Black arrow indicates treatment start. [file ar3329-S2.PDF]

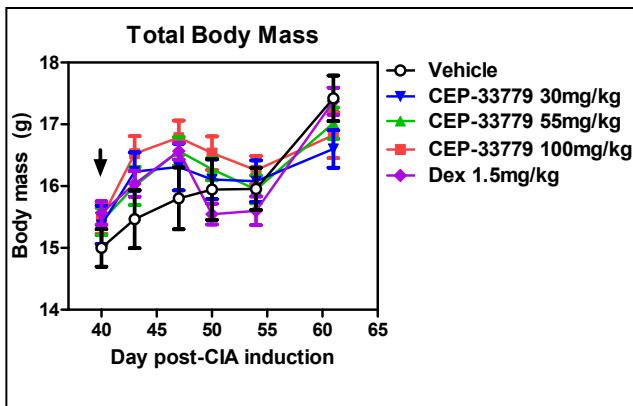

**Figure S3.0.**

Supplement: Additional file 3 — Total body mass of CEP33779-treated CIA model mice. Graph shows means ± SEM of total body mass of CIA mice. Female DBA/1 mice were treated with CEP-33779 p.o., b.i.d., Dex at 1.5 mg/kg three times weekly, and vehicle PEG400 + 1% DMSO p.o., b.i.d. Study size shows n ≥ 10 mice per group and n ≥ 5 mice for individual assays. Black arrow indicates treatment start. [file ar3329-S3.PDF]

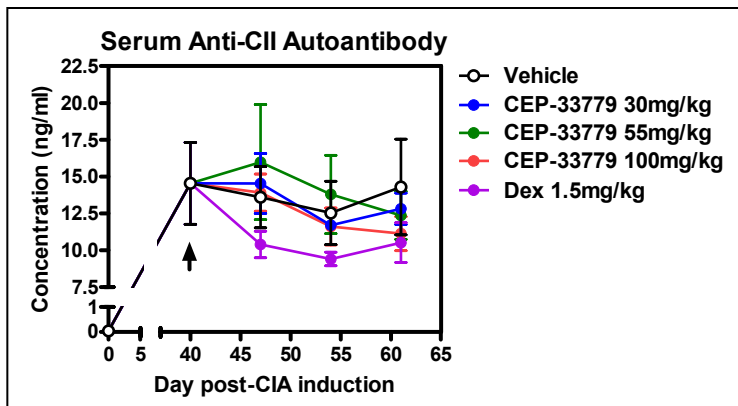

**Figure S4.0.**

Supplement: Additional file 4 — No change in serum anti-CII autoantibodies in CIA mice treated with CEP-33779 over time. Female DBA/1 mice were injected with purified CII in CFA i.d., then boosted with CII in IFA s.c. on day 21 followed by a day 28 LPS injection i.p. to induce CIA. Mice that scored 1 or better for each paw were considered arthritic and entered into the study. Treatments started after several days of full arthritis. CEP-33779 was administered p.o., b.i.d., throughout the remainder of the experiment. Dex was administered i.p. at 1.5 mg/kg three times weekly, and vehicle PEG400 + 1% DMSO was administered p.o., b.i.d. Serum testing using an anti-CII autoantibody enzyme-linked immunosorbent assay (ELISA) is described in Materials and methods and elsewhere [18]. No statistical significance was observed for any group compared to vehicle using two-way ANOVA. Serum dilution used was a five fold dilution for all samples, with the diluted sample shown. Graph shows means ± SEM, n ≥ 10 mice per group tested, with serum collected at weekly intervals and stored at -80°C until tested. Study size shows n ≥ 10 mice per group and n ≥ 5 mice for individual assays. Black arrow indicates treatment start. [file ar3329-S4.PDF]
